# 

**Published:** 1844-01-13

**Authors:** 


### INDEX TO VOLUME VII.

Antagonism of various diseases, 12
Abdominal surgery, 14
Apoplexy and paralysis, paraplegia, 17
Aneurism of the aorta, &c. 29
Aorta and pericardium, wound of the, 33
Anchyloblepharon and symblepharon, 50
Anatomy and physiology in the year 1842–'3, report on the progress of, by James Paget, 44, 55, 66, 79, 89, 104. 117, 128
Arsenic, method of detecting, by means of copper, 47; in ascites, 192; improved method for the detection of, 248
Abdomen, severe injury of, with protrusion of the intestines, followed by recovery, 49
Asthma, nitrate of potass in, 49
Antagonism of typhoid and intermittent fevers, 58
Absorption, 67
Animal life, organs of, 80
Admission of air into a vein during an amputation, case of alarming syncope from, 81
Animalcules in the intestinal canal, 81
Anatomical researches on the excretory ducts of the lachrymal gland, 82
Alkaline saliva, 83
Aneurism by anastomosis, 39; aortic, 62; notes of three cases of, 85; fibrous tumour mistaken for, 108; interesting case of, near the origin of the aorta, 109; result of the operation for, 136
Alison's outlines of pathology and practice of medicine, 115
Andral's pathology of hæmatology, 115
Aberration in the appreciation of colors, 120
Age of puberty in girls, 128
Albuminuria, or Bright's disease, 129
Alcoholic lotion, on the use of the, in phthisis pulmonalis, 131
Automaton speaker, 149
Adelon, Beclard and Berard's cyclopædia of practical medicine, 175
Amussat, Andral and Boivin's dictionnaire des etudes medicales pratiques, 175
Albany county medical society, 188
Abscess of the neck, scrofulous, 192; of the heart, 203
Aneurism, carotid, 316; of the external iliac, 227
Aconite, death by poisoning with the leaves of, 225
Amaurosis cured by inoculation with the sulphate of strychnia, 226
Anus, artificial, in the left lumbar region, 250
Amputation of the entire scapulum, 251
Atlee's introductory lecture, 298
Artesian wells at Naples, 300
Abortion, artificial, 223

Bibliographical notices, 5, 19, 30, 42, 52, 63, 76, 88, 101, 115, 125, 136, 148, 175, 186, 197, 209, 221, 237, 246, 270, 283, 297, 303
Brain, fungus of the, 11; softening of the, 41
Boils, 11
Blood of a living dog, filariæ in the, 12
Bilocular uterus, 23
Barker, Dr.'s, case of double brain, 23
Bubo in the pelvis, 35
Blood, 44
Brun's manual of the general anatomy of man, 54
Blockley almshouse and Philada- hospital, census of the, 61
Bark, on yellow, 69
Burdett, Sir Francis, death of, 80
Blood-letting as a diagnostic, 83
Biliary calculus, case in which one of unusual size was safely passed, 87
Blephoroptosis, operation for the cure of, 97
Burns, cure of, 120
Bright's disease, or albuminuria, 129
Bouchardat's annuaire de therapeutique, &c. 136
Blondlot's traite analitique de la digestion, 148
Belladonna in tetanus, 168
Bowel, case of intussusception of, 169
Botanic physicians of Rochester, 191
Betton's translation of Moreau's treatise on midwifery, 197
Boston Medical and Surgical Journal, 201
Bartlett's essay on the philosophy of medical science, 246
Brain, concussion of, 249
Bronchi, foreign bodies in, 275
Bromide of iron, preparation of, 300
Beeswax, yellow, on the bleaching of, 300
Bulletin of Medical Science, 165

Clinical lectures, 2, 3, 17, 28, 39, 50, 61, 74, 97, 114, 124, 134, 139, 151, 160, 161, 171, 181, 193, 207, 217, 233, 242, 257, 265, 277, 289, 301
Cancrum oris, 2
Closing fistulous orifices of the bony palate by a plastic operation, possibility of, 13
Chorea, 18
Clinical instruction, 21
Churchill's medical report of the Western Lying-in Hospital, 19; on inflammation and abscess of the uterine appendages, 30; notes on ovariotomy, 198; diseases of females, 259
Camphor on the sexual organs, 24
Chronic diarrhœa and dysentery, 28
Cardiac disease, 28
Cooper on the anatomy and surgical treatment of abdominal hernia, 31
Chomel on the diagnosis of pneumonia, 34
Cæsarian section successfully performed; both mother and child saved, 35
Chest, contracted, from chronic pleurisy, 41
Circulation, 55
Census of the Blockley almshouse and Philadelphia hospital, 61
Chapman's lectures on the diseases of the thoracic and abdominal viscera, 63; on eruptive fevers, &c., 283
College, Pennsylvania, 66; Jefferson medical, 66
Controversy, orthopedic, in Paris, 67
Calomel, utility of, in typhoid fever, 69
Case of stricture of the ileum, and perforation of the appendix vermiformis cæci, 73
Caries of the acromion process, 74
Case of the removal of a diseased ovarium, 78
Calculus, biliary, case in which one of unusual size was passed, 87
Congenital inguinal hernia, on the treatment of—operation, 94
Chailly's practical treatise on midwifery, 101
Compound fracture of the skull, 106
Children, on the temperature in, 120
Cure of burns, 120
Coloured saliva, 121
Cashew nut, poisoning by, 133
Cheiloplastic operation by Prof. Mutter, 158
College of Physicians of Philadelphia, annual meeting of, 167
Costello's cyclopædia of practical surgery, 175
Copland's dictionary of practical medicine, 175, 259
Chadwick's report on the results of an inquiry into the practice of interments in towns, 186
Chancre in the urethra, 167; tanning a, 202
Catheterism, 168
Chloride of magnesium, 180; of sodium in disease of the eye, 287
Cancer, removal of, by the knife, 180; essential pathology of, 191; treatment, 216
Cincinnati Journal of Health, 188
Corrosive sublimate, poisoning by, 203
Cachexia, cancerous, 225
Cataract, singular formation of, 226; spontaneous cure of, 226
Carcinoma of the breast and liver, 240
Cooper, the late Sir Astley, 263
Callus, partial solution of, from low living, 264
Carpus, backward dislocation of, 264
Carbon eliminatedby plants, 276
Calvarium, long issue in the, 276
Creasote in nævus maternus, 287
Cruveilhier's anatomy, 299
Cataract, spontaneous cure of, 300
Consumption in Illinois, 302

Dislocation of the os femoris, 2
Double brain, Dr. Barker's case of, 23
Diagnosis of pneumonia, M. Chomel on the, 34
Dunglison's human physiology, 42; dictionary of medical science, 76; practice of medicine, 115; on human health, 187; medical student, 239
Dropsy, ovarian, operation for, 48; renal, 289; pathological curiosity in a case of, 292
Darrach's anatomy of the groin, 64
Digestion, 66
Different kinds of opium, 70
Death of Sir Francis Burdett, 80; from ardent spirits, 240
Dropsy, fatal, after scarlatina, 107
Death of Sir Henry Halford, 120; after parturition, 284; of Dr. Forrey, 271
Diseases of the eye, 126; of the tibia, 156; of the bones and joints, 242
Deformity from a dislocation of the head of the humeris, 136
Druitt's principles and practice of modern surgery, 188
Dispensary, Philadelphia, 202
Dental pulp, Dr. J. D. White on the treatment of, 241
Diabetes, treated by alkalies, 250
Daguerreotype process, modification of, 300
Diseases of the respiratory organs, 301

Editorial remarks, 6, 21, 31, 43, 54, 65, 77, 104, 116, 126, 137, 149, 165, 178, 188, 200, 210, 239, 247, 260, 271, 283, 299, 304
Employment of belladonna in the treatment of phimosis and paraphimosis, M. de Mignot on the, 24
Extirpation of the salivary glands in animals, 24
Encysted hydrocele, Mr. Liston on, 34
Extraordinary case of varix, 34
Erysipelas, 41
Encysted stone, case of, 46
Eye, punctures of the, 46; diseases of the, 126; images reflected in the, 142
Effects of a large dose of quinine administered by mistake, 60
Enlargement of the glands at the cardiac orifice of the stomach, starvation from, 70
Extirpation of an ovarian cyst, case of, 78
Excretory ducts of the lachrymal gland, anatomical researches on, 82
Excision of the whole lower jaw, 96
Extensive meloplastic operation, 111
Epidemic ague, or fainting fever of Persia, 119
Effects of drainage on human life, 142
Epilepsy, 51; statistics of, in Philadelphia hospital, 146
Extraction of foreign bodies, 172, 181
Encyclopadisches worterbuch des medicinischen wissenschaften, 175
Editorial courtesy, 178
Epidemic erysipelas in Delaware county, Pa. Dr. Young's account of, 206
Elephantiasis græcorum endemic in certain parts of Norway, 215
Exomphalus, new mode of reducing, 226
Electric current, means of avoiding, 228
Empyema, singular case of, 240
Ergot of rye, new mode of preserving, 249
Erysipelatous inflammation of the scalp, 301

Fungus of the brain, 11
Filariæ in the blood of a living dog, 12
Fungus hæmatodes, supposed case of, combined with melanosis, 25
Fibro-cellular tissue, 44
Face-ache, 47
Females, born co-twins with males, alleged infecundity of, 47
Forceps, Bond's placental, 54
Face and head, ticdoloureux of the, 69
Fæcal incontinence, open anus, in certain forms of, 82
Facial neuralgia, 106
Fatal dropsy after scarlatina, 107

Fibrous tumour mistaken for aneurism, 108
Formation and structure of the membranes, 117
Foy's traite de matiere medicale, 136; formulaire des medicine praticiens, 136
Forbes', Tweedie, and Conolly's cyclopædia of practical medicine, 175
Females, proper age to marry, 178
Formula for precipitated chalk, 179
Fistula in ano, 193, 207
Fracture, compound, of both tibia and fibula, 273
Fevers, intermittent, 276
Fiske fund prize questions, 276
Fever, typhoid, 277, 289
Fracture of the skull, 307

Guy's principles of forensic medicine, 6
Grinder's lungs, 11
Gout—cold water treatment—translation to the heart and stomach—death, 36; frequent relapse, and ultimate recovery, 36
Gastritis, chronic, 40
Gastro-enteric affection, anomalous, 61
Gravid uterus, on the nerves of the, 70
Graduates at Jefferson Medical College, March, 1844, 72
Glanders, case of death from, 83; communicated by the bite of a horse, 275
Graduates at the University of Pennsylvania, April 4, 1844, 83
Generation, 105
Girls, age of puberty in, 128; changes in age—varia, 128
Germany, medical institutions of, 224
Gangrene of the lung, 240
Gestation impeded by encysted dropsy, 248
Greenhill's Sydenham, 271

Hysteria, 22
Heart, rupture of the, and sudden death, 25
Health, influence of employments upon, 36, 58; in tobabco manufactories, 252; in Philadelphia, 187
Hare's statistical report of 190 cases of insanity, 42
Hermaphroditism, theory of, 47
Hydrocephalus, chronic, 50, 62
Hydrocele, new method for the radical cure of, 60
Head, injuries in the, state of the pupils in, 83
Hemiplegia, 3, 51; following ligature of the common carotid, 106
Hydropic cachexia, 51, 74
Harelip, operation for, 119
Halford, Sir Henry, death of, 12
Humerus, malignant disease of the, 130
Hæmorrhage after parturition, 132; of the liver, 202; fatal, from perforation of the aorta, by false teeth, 215; spontaneous from the bulb of the eye, 226; from leech bites, means of arresting, 263; obstinate, following the extraction of a tooth, 275
Hypertrophy of the heart, and open foramen ovale, without cyanosis, 133
Heating power of the sun, 156
Hydropathy, 181, 211
Hope, Dr., death of, 190
Hotel Dieu 50 years ago, 192
Health of Philadelphia, 210
Hydatids, abdominal, 215
Hooker's dissertation on the respect due to the medical profession. 223
Hecker on the epidemics of the middle ages, 237
Hernia cerebri, 240
Homœopathy at a discount, 252
Head, Dr. C. Huston's case of injury of, from a fall, 265
Hæmoptysis, 289

Impostors, more mesmeric, 13
Intermittent fevers, 28
Influence of employments upon health, 36, 58
Infecundity of females born co-twin with males, alleged, 47
Insanity at childbirth, tendency to, 70
Infrequency of phthisis in marshy districts, 71
Intestinal canal, animalcules in, 81
Intestine, strangulation of, fatal case of, 87
Ilium, perforation of, commencing on the peritoneal surface, 95
Iron, preparations of, dangerous in certain forms of chlorosis, 96
Interesting case of aneurism near the origin of the aorta, 109
Intelligence, Parisian, 118
Iodide of potassium in the latter stages of pneumonia, 145; of mercury in syphilitic caries, 252
Ileus from the turning or twisting of a bowel on its axis, 145
Intussusception of the bowel, 169
Iodine, tincture of, 226
Ipecacuanha, Dr. Cormack on, as a counter-irritant, 248
Inflammation, pseudo-membranous of the bladder, 251
Idiots, management of, 251
Insane in Canada, 271
Insanity caused by excessive drinking, 275
Introductory lectures, 283, 303
Imbibition, diseases of, 278

Jefferson Medical College, graduates at, March, 1844, 72
Jaw, lower, excision of the whole, 96

Leucorrhœa, 2
Lectures, introductory, 7
Lupus, case of, 29
Liston, Mr., on encysted hydrocele, 34
Lacerated wounds, 35
Labour, one hundred and seven cases of, occurring in the lying-in department of Philadelphia Hospital (Blockley), statistical report of, 37; process of, 204; complicated with recto-vaginal hernia, 231
Lactation, 117
Lepra in Italy, 123; vulgaris, 202
Lee on the theory and practice of midwifery, 177
Ludlow's manual of examinations upon anatomy, physiology, &c. 177
Laudanum, electricity in cases of poisoning by, 179
Liver, hemorrhage of the, 202
Lancet, London, gross deception in the reprint of the, 205; do, 301
Living child, 39 days after quickening, 214
Lever's practical treatise on organic diseases of the uterus, 221
Louis' researches on phthisis, &c., 237
Leech, European, raising of, 242
Lunacy, French commission of, 262
Larynx, foreign body lodged in, 274
Leprosy, Canada, 305
Lectures, by
Prof. Huston, 1
Prof. Dunglison, 3, 17, 28, 40, 51, 61, 74, 97, 265, 277, 289, 301
Prof. Mutter, 3, 29, 39, 50, 124, 134
Prof. Pancoast, 99, 280, 292
Prof. Bache, 114, 166, 171
Sir C. Brodie, 161, 172, 181, 193, 207, 217, 233, 257, 268
Robert Liston, Esq., 242

Muscles of the face, operation for the contraction of, 1
M. de Mignot on the employment of belladonna in the treatment of phymosis and paraphymosis, 24
Mesmeric imposters, 13
Muscular tissue 45
Method of detecting arsenic, by means of copper, 47
Malignant diseases, treatment of, 60
Method, new, for the radical cure of hydrocele, 60
Magendie's elementary treatise on human physiology, 64
Manual operations, love for, 70
Microscopic examination of the tartar of the teeth, 71
Medical graduates of the present season, 89
Menstruation, vicarious, following an accident to the finger, 107; facts relating to the period at which it ceases in women, 288
Meloplastic operation, extensive, 111
Membranes, formation and structure of the, 117
Men, physical history of, 117
Malignant diseases of the humerus 130,
Midwives of Greece, and their remedies, 132
Mental emotion, effects on the physical frame, 167
Mercury and quinia, new salt of, 179
Magnesium, chloride of, 180
Mackintosh's principles of pathology and practice of medicine, 209
Mississippi valley, diseases of the. 201
Medical examinations and licenses in Cuba, 211
Malformation of the heart in a child, 217
Meig's, Dr. C. D., case of malformation of the heart, 217; case of labor complicated with recto-vaginal hernia, 231, successful removal of, by ligature, 240; case of twins, &c., 241; extirpation of the uterus for complete inversion, 268
Medical institutions of Germany, 224
Moxas of marmoral 251
Matico, as a styptic and astringent, remarks on. 262
Moliform-fibro-cartilaginous tumour, Dr. Pancoast's care of, successfully extracted, 272
Medical classes, 6, 271; Society, 21, 43; Schools, 54; institute of Louisville, 55; instruction in Philadelphia during the summer months, 65
Medical student's Temperance Society, 11; schools of Philadelphia, 260
Meeting of medical superintendents of hospitals for the insane, 261
Mal-practice, 261
Mercurial stomatitis, 291
Mesmerism, triumphs of, 306
Medical mixtures in styptics, 306
Membranes, false, formation of within the bladder, from cantharides blisters applied to the skin, 308

Nitrate of silver in ophthalmia, 26; of potash in spasmodic asthma, 180
New test for strichnine, 43
Neuralgia of the urethra, 69; facial, 274
Naphtha, trial and failure of, 71
Nutritive substances, transformation of, 79
Notes of three cases of aneurism, 85
Nervous system, 89
Necrosis of a portion of the frontal and parietal bones—purulent collection in the right hemisphere of the brain—hemiplegia of the right side, 93
New York hospital and Bloomingdale asylum, 103
Naphtha in phthisis, 106
Neuralgia, facial, 106; neuralgia, 168
Nose, restoration of the right ala of the, 136
Neapolitan phlebotomist, 155
Nux vomica in neuralgia, 108
Necrosis of the lower jaw, 213
Non-schirrous tumours of the breast, 233
Nævus, superficial, 251
New Orleans, health of, 278; mortality of, 273
Notice of three children at a birth, 288
New York Journal of Medicine, 116; do, and Chailly's midwifery, 165, 228
Necro-pneumonia, 301
Nervous aphonia cured by electricity, 308

Operation for contraction of the muscles of the face, 1; for false anchylosis of the jaw, 280; for caries of the os calcis, 282; for caries of the phalanges of the great toe, 282
Os femoris, dislocation of, 1
Organization of Paris hospitals, 10
Ophthalmia, nitrate of silver in, 24
Ovarian dropsy, operation for, 48
Obstetric department of the Philadelphia dispensary, report of the 61
Orthopedic controversy in Paris, 67
On yellow bark, 69
On the nerves of the gravid uterus, 70
Opium, different kinds of, 70
Ovarium, case of the removal of a diseased, 78
Ovarian cyst, case of extirpation of, 78
Organs of animal life, 80
Open anus in certain forms of fæcal incontinence, 82
Osteosarcoma and encephaloid tumour of the fore-arm, 120
Oxalic acid diathesis, 154
Obstructions of the pulmonary arteries, 155
Obstetric case, complicated with encysted tumour of the uterus, 167
Ovariotomy, 77
Otto, Dr., death of, 166
Obliteration of the urethra, &c., 281

Pancoast's successful operation for the contraction of the muscles of the face, 1; treatise on operative surgery, 101
Puerperal fever, 2
Perforated intestine, 3
Pleuropneumonia, 3
Pathology of phlegmasia dolens, 8
Paris hospitals, organization of, 10
Pregnancy, case of, while the uterus was external to the abdomen 12; and cancer uteri, 180
Possibility of closing fistulous orifices of the bony palate by a plastic operation, 13
Peters's review of Caldwell's pamphlet, 20
Pregnancy two years after cessation of the menses, 21

Pregnancy in the vagina, 23
Pneumonitic abscess, 28, 63
Peritoneal section for the removal of two diseased ovaria, Dr. J. L. Atlee's case of, 31
Pelvis, bubo in the, 35
Pessaries, 43
Paget's report on the progress of anatomy and physiology in the years 1842–'3, extract from, 44, 55, 66, 79, 89, 117, 128
Pregnancy, sign of, 45
Punctures of the eye, 46
Parts, rapid re-union of, 48
Potass, nitrate of, in asthma, 49
Paris' pharmacologia, 52
Piles, nitric acid for, 60
Pharyngitis, &c., 61, 291
Phthisis in marshy districts, infrequency of, 71; influence of marshy exhalation on, 232
Psoriasis and lepra, treatment of, 83
Portia dura, paralysis of, in an infant, 94
Perforation of the ilium, commencing on the peritoneal surface, 95
Porrigo decalvans, vegetable origin of, 96
Preparations of iron dangerous in certain forms of chlorosis, 96
Philadelphia hospital—women's lunatic asylum, 100
Phthisis, naphtha in, 106
Paralysis of the hand and wrist cured by strychnine, 108
Physical history of man, 117
Parisian intelligence, 117
Philadelphia dispensary, quarterly report of the obstetric department of the, 61, 123
Polypus uteri, 130
Phthisis pulmonalis, on the use of the alcoholic lotion in, 131
Parturition, hæmorrhage after, 132
Poisoning by the cashew nut, 133; by corrosive sublimate, 135, 203, 287; by the leaves of aconite, 225
Prolapsus ani, 134
Polypi of the nose, diseases which are sometimes mistaken for, 139
Pneumonia, iodide of potassium in the latter stages of, 145
Pulmonary arteries, obstructions of the, 155
Pathological specimens, preservation of, 156, 249
Peritonitis terminating fatally, 157
Portia dura, division of, 191
Pettigrew on the superstitions connected with the history and practice of medicine and surgery, 197
Presentation of the head and foot, 206
Provincial Medical and Surgical Association, (England), 247
Pleuro-pneumonia, 228; cure of, by acute mania, 263
Pancoast's case of molliform fibro-cartilaginous tumour, 272
Potassæ, liquor, a test for diabetic urine, 276
Punch's physiology of the London medical student, &c., 298
Philadelphia Medical Association, 178; Society, 299
Pulmonary phthisis, 303
Professional courtesy, 304

Quinine, effects of a large dose of, administered by mistake, 60; in epilepsy, 251
Quinia and mercury, new salt of, 179
Quackery at a discount, 239

Rilliet and Barthez's Clinical and Practical Treatise on the diseases of children, 19
Rupture of the heart and sudden death, 23
Report of the Surgeon general of the U. S. Navy, 26; of the directors and superintendent of the Ohio lunatic asylum, 42; of the Pennsylvania hospital for the insane, 53; of the inspectors of the eastern state penitentiary, 88; of the eastern asylum, of Williamsburg, Va., 125; on the state of the asylum for the relief of persons deprived of their reason, 125; of English lunatic asylums, 297
Rubeola, urtication in the treatment of, M. Trousseau on, 35
Reunion of parts, rapid, 48
Rheumatism, treatment of, 48
Respiration, 55
Report of the obstetric department of the Philadelphia dispensary, 61
Royal Academy of Medicine, Paris, 92; of sciences of do, 156
Rupture of the stomach, &c., 121; of the heart, 276
Remedies of the midwives of Greece, 132
Rhinoplastic operation, professor Mutter's, 147
Remedies, antipsoric, 180
Raciborski, de la puberte et de l'age critique chez la femme, &c, 181
Royal college of surgeons in England, 202
Retinitis, acute, caused by the use of the microscope, 204
Reports, clinical lectures and, 247, 271
Rectum, foreign bodies in, 263

Stone masons lungs, 11
Surgery, abdominal, 14
Salivary glands in animals, extirpation of, 24
Sexual organs, influence of camphor on, 24
Supposed case of fungus hæmatodes, combined with melanosis, 25
Surgeon general of the United States Navy, report of the, 26
Statistical report of one hundred and seven cases of labour, occurring in the lying-in department of the Philadelphia Hospital (Blockley), 37
Singular case of neurosis, 39
Strychnine, new test for, 43
Sign of pregnancy, 45
Stone, case of encysted, 46
Spina Bifida, 47
Severe injury of the abdomen, with protrusion of the intestines, followed by recovery, 49
Smith's anatomical atlas, 53, 210, 222, 239
Scrofulosis, 64
Starvation from enlargement of the glands at the cardiac orifice of the stomach, 70
Stricture of the ileum, and perforation of the appendix vermiformis cæci, case of, 73
Stoke's diagnosis and treatment of diseases of the brain, 76
Syncope from the admission of air into a vein during an amputation, alarming case of, 81
State of the pupils in injuries of the head, 83
Strangulation of the intestine, fatal case of, 87
Special senses, 104
Skull, compound fracture of, 106
Strychnine, paralysis of the hand and wrist cured by, 108
Stomach, rupture of the, &c., 121; cancer of the, 307
Strumous conjunctivitis, 123
Saliva, coloured, 131
Sydenham Society, 31, 134, 200, 260
Sprain of the ankle-joint, 136
Stricture, urethra, obstinate cases of, 156
Sun, heating power of the, 156
St. Hilaire, Geoffrey, death of, 190
Surgery, progress of, in China, 191; important points of, 257, 268
Scrofulous abscess of the neck, 192
Schirrous of the thyroid glands, 214; of the uterus, 215; of the breast, 217
Source of reproduction after the death of the shaft of a long bone, 216
Spleen, excision of the, 240
Seasoning, 240
Sea-sickness, a remedy in certain cases of jaundice, 250
Spinal distortion, 251
Sectional medicine, Dr. F. W. Bullitt on, 253
Summary of the transactions of the College of Physicians, 283
Scarlatina, 289; 301

Tissue, fibro-cellular, 45; muscular, 45
Theory of hermaphroditism, 47
Treatment of rheumatism, 48
Typhoid and intermittent fevers, antagonism of, 58; fever, 97; 301
Treatment of malignant diseases, 60
Tic douloureux of the face and head, 69
Tendency to insanity at childbirth, 70
Trial and failure of naphtha, 71
Tartar of the teeth, microscopic examination of, 71
Transformations of nutritious substances, 79
Tweedie's cyclopedia of practical medicine, 88
Treatment of congenital inguinal hernia—operation, 94
Tuberculosis of the lung, 98
Tuberculous subjects, deafness of, 103
Tunica vaginalis testis, obliteration of the cavity of, 119
Temperature, on the, in children, 120
Transactions of the New York State Medical Society, 138
Therapeutics, report on some late statements in, 142
Transylvania University, 150
Tongue, diseases of the, 140: tumour of the, 39
Tibia diseases of, from injury, 156
Typhoid diseases, new diagnostic signs in, 167
Tetanus, belladonna in, 168
Teeth, on the extraction of, and the instruments in use for that purpose, 159; Dr. J. F. B. Flagg's remarks on the extraction, 169
Todd's encyclopedia of practical medicine, 175
Twins, footling presentation of, 191
Thyroid glands, schirrous of the, 214
Twins, case of, with locking of the fore arm of one of them, 241
Transposition of the heart, stomach, liver and spleen, &c., 264
Tumour, carcinomatous of the neck, 249; extensive, 134; of the lower eyelid, 135; deep-seated in the perineum, 281
Transudation, pulmonary, 278; encephalic, 279
Tubercular deposits, 302

Uterus, bilocular, 23; encysted tumour of the, 167
Urtication, in the treatment of rubeola, M. Trusseau on, 35
Urethra, neuralgia of the, 69; chancre in the, 167
Utility of calomel in typhoid fever, 69
University of Pennsylvania, graduates at, April 4, 1843, 83
Uteri, polypus, 130
Urethral stricture, obstinate case of, 156
University of King's College, 211
Ulceration and perforation of the aorta, Dr. Upshur's case of, 230; of the larynx, 251
Ulceration and perforation of the intestinal canal, 301; of the throat, 302

Various diseases, antagonism of, 12
Vagina, pregnancy in the, 23
Varix, extraordinary case of, 34
Vomitings of early pregnant women, a remedy for, 71
Vegetable origin of porrigo decalvans, 96
Vicarious menstruation following an accident to the finger, 107
Vesalius, statue of, 120
Variola, chloruretted lotions in, confluent, 179
Vulgaris, lepra, 202
Vaccination, protective influence of, 274
Vaginal hysterotomy and criminal abortion, 77

Watson's lectures on the principles and practice of physic, 5
Wound of the aorta and pericardium, 33 of the heart, 307; case of recovery from, with hernia of the lung, 308
Wounds, lacerated, 35; of the ear, 50
Women's Lunatic Asylum—Pennsylvania Hospital, 100
Warning to mothers, 119
Western Journal of Medicine and Surgery, 201
Worthington, Dr. H., case of presentation of the head and foot, 206
Wilson's system of human anatomy, 209
Wattman's sicheres heilverfahren, &c. 210
Wakley, Mr., vote of thanks to, 262

Yale College, 31
Yandell's rejoinder to McDowell, 270

Zinc, valerianate of, 180; a powerful antispasmodic, 308

